# The VA Normative Aging Study: The Aging of a Longitudinal Study of Aging

**DOI:** 10.1093/geroni/igaf122.1457

**Published:** 2025-12-31

**Authors:** Avron Spiro

**Affiliations:** Boston University - Chobanian & Avedisian School of Medicine, Waban, United States

## Abstract

In 1963, the Normative Aging Study (NAS) was initiated at the Boston VA Outpatient Clinic. Over the next several years, 2280 men, mostly veterans, were enrolled into a life-long study of aging. To better understand the distinction between aging per se vs. the progression of disease, these men were selected for good health, regardless of age. The intention was to examine, over their lifespan, the interplay between aging and the development and progression of disease. Men returned to the clinic every few years for examinations and completed periodic surveys that assessed their health, disease, anthropometry, and health behaviors. In this talk, we will discuss the origins of the NAS and its place in the first wave of longitudinal studies of aging (e.g., Framingham, Baltimore, Duke). We will then focus on the growth of the psychosocial research program at NAS, which was nascent in the initial decades but expanded rapidly during the 1980’s. This program examined social support, stress and coping, mental health, personality and cognition. We will also mention recent work in this deeply phenotyped study that focuses on epigenetics and its role in psychosocial aging and health. In concluding, we offer some observations on the complexities of establishing and maintaining longitudinal studies and their contributions to the study of aging and development.

